# Eribulin Synergistically Increases Anti-Tumor Activity of an mTOR Inhibitor by Inhibiting pAKT/pS6K/pS6 in Triple Negative Breast Cancer

**DOI:** 10.3390/cells8091010

**Published:** 2019-08-30

**Authors:** Wei Wen, Emily Marcinkowski, David Luyimbazi, Thehang Luu, Quanhua Xing, Jin Yan, Yujun Wang, Jun Wu, Yuming Guo, Dylan Tully, Ernest S. Han, Susan E. Yost, Yuan Yuan, John H. Yim

**Affiliations:** 1 Division of Surgery, Beckman Research Institute, City of Hope National Medical Center, 1500 East Duarte Rd., Duarte, CA 91010, USA; 2 Department of Medical Oncology and Molecular Therapy, Beckman Research Institute, City of Hope National Medical Center, 1500 East Duarte Rd., Duarte, CA 91010, USA; 3 Department of Comparative Medicine, Beckman Research Institute, City of Hope National Medical Center, 1500 East Duarte Rd., Duarte, CA 91010, USA

**Keywords:** TNBC, eribulin, PI3K/AKT/mTOR, everolimus, combination, synergy

## Abstract

Unlike other breast cancer subtypes, patients with triple negative breast cancer (TNBC) have poor outcomes and no effective targeted therapies, leaving an unmet need for therapeutic targets. Efforts to profile these tumors have revealed the PI3K/AKT/mTOR pathway as a potential target. Activation of this pathway also contributes to resistance to anti-cancer agents, including microtubule-targeting agents. Eribulin is one such microtubule-targeting agent that is beneficial in treating taxane and anthracycline refractory breast cancer. In this study, we compared the effect of eribulin on the PI3K/AKT/mTOR pathway with other microtubule-targeting agents in TNBC. We found that the phosphorylation of AKT was suppressed by eribulin, a microtubule depolymerizing agent, but activated by paclitaxel, a microtubule stabilizing agent. The combination of eribulin and everolimus, an mTOR inhibitor, resulted in an increased reduction of p-S6K1 and p-S6, a synergistic inhibition of cell survival in vitro, and an enhanced suppression of tumor growth in two orthotopic mouse models. These findings provide a preclinical foundation for targeting both the microtubule cytoskeleton and the PI3K/AKT/mTOR pathway in the treatment of refractory TNBC.

## 1. Introduction

Triple negative breast cancer (TNBC) accounts for 12–17% of all breast cancers, and is characterized by a poor overall and relapse-free survival [1]. Unlike hormone receptor positive tumors and tumors with Her2-neu overexpression, patients with TNBC have worse outcomes after chemotherapy and have an unmet need for targeted therapy [2,3]. As such, efforts to profile TNBC tumors have identified the PI3K/AKT/mTOR pathway as a potential therapeutic target [4,5,6].

The PI3K/AKT/mTOR pathway is a key signal transduction pathway that mediates cellular responses to growth factors [7,8,9]. This pathway affects many cellular functions, including cell survival, cell proliferation, and apoptosis [10]. Patients with TNBC often have high levels of AKT expression and activation of the PI3K/AKT/mTOR pathway [2,4,11,12]. Treatment targeting the PI3K/AKT/mTOR pathway in patients with alterations in the PI3K/AKT/mTOR pathway resulted in significantly better outcomes in both treatment naïve and previously treated patients [2,12]. However, an mTOR inhibitor, when used alone, can induce increased levels of p-AKT via a negative feedback loop, leading to resistance of cells to mTOR inhibitors [13,14]. Novel combinations are urgently needed to effectively target PI3K pathway alterations in patients that progress on therapy or fail to respond [9,10,15,16].

Microtubule-targeting agents have been used with success to treat TNBC [1,5,17]. Eribulin mesylate (E7389), a synthetic macrocyclic analogue of the marine sponge natural product halichondrin B [18,19], suppresses mitosis by directly binding to microtubule ends, resulting in the inhibition of microtubule growth and formation of tubulin aggregates. This leads to abnormal mitotic spindles that cannot pass the metaphase/anaphase checkpoint, effectively inducing G2-M cell cycle arrest [20,21,22,23,24,25,26]. Eribulin has demonstrated a potent anti-tumor activity against a wide range of tumor cells both in vitro and in vivo. Eribulin can also be combined effectively with other anticancer agents [27,28]. Eribulin has been approved for the treatment of TNBC in heavily pretreated patients [29].

Breast cancer cells can eventually become resistant to targeted therapy or chemotherapy, despite initial response to the treatment [30,31,32]. Activation of the PI3K/AKT/mTOR pathway contributes to the resistance to anti-cancer agents, including microtubule-targeting agents. In this study, we investigated the effect of eribulin and a mTOR inhibitor, either alone or in combination, on the PI3K/AKT/mTOR pathway and tumor growth in TNBC. Our results demonstrate that eribulin, unlike paclitaxel, potently decreases the expression of p-AKT in TNBC. Dual treatment of eribulin and the mTOR inhibitor results in a synergistic suppression of cell survival in a number of TNBCs in vitro and an enhanced suppression of tumor growth in two TNBC mouse models.

## 2. Materials and Methods

### 2.1. Reagents

Eribulin was kindly provided by Eisai Co. Ltd. (Tokyo, Japan). Everolimus (RAD001), BKM120, and BEZ235 were kindly provided by Novartis (Basel, Switzerland). Antibodies against p-AKT (S473), AKT, p-S6K, S6K, p-S6, S6, and GAPDH were obtained from Cell Signaling Technology (Danvers, MA, USA). Anti-β-actin was obtained from EMD Millipore (Billerica, MA, USA).

### 2.2. Cell Viability Assays

The human breast cell lines BT549, Hs578T, MDA-MB-231, and MDA-MB-468 were obtained from American Type Culture Collection (Rockville, MD, USA). Cells were cultured in RPMI 1640 medium (Mediatech Inc., Manassas, VA, USA) for BT549 and 4T1 cells, in 1:1 DMEM/F12 (Gibco, Thermo Scientific, Waltham, MA, USA) for MDA-MB-468 cells, and in DMEM medium (Mediatech Inc., Manassas, VA, USA) for MDA-MB-231 and Hs578 cells. Culture media were supplemented with 10% fetal bovine serum (Atlanta Biologicals, Norcross, GA, USA) and 1% penicillin/streptomycin (Gibco, Thermo Scientific, Waltham, MA, USA).

Cells (4000 per well) were plated in a 96-well plate format in 100 μL growth medium. Cells were treated with dimethyl sulfoxide (DMSO) or drugs the next day at the indicated concentrations and incubated for an additional 3 days. Viable cells were determined by the 3-(4,5-dimethyl-thiazol-2-yl)-2,5-diphenyltetrazolium bromide (MTT) assay (Promega, Madison, WI, USA) [33]. After treatment, the media were removed and MTT dye was added to each well and incubated for 4 h according to the manuscripter’s instruction. The resulting formazan crystals were dissolved in DMSO after removal of the media. Absorbance was read at 570 nm. The half maximal inhibitory concentration IC_50_ was determined using the Calcusyn software package (Biosoft, Ferguson, MO, USA).

### 2.3. Combination Index (CI)

The Chou-Talalay method was used to calculate the combination index (CI) with the Calcusyn software package (Biosoft, Ferguson, MO, USA) [34]. CI < 1 indicates synergism, CI > 1 indicates antagonism, and CI = 1 indicates an additive effect.

### 2.4. Western Blot

Cells were treated with DMSO or drugs in complete medium at the indicated concentrations and times, washed with cold phosphaste-bufferd saline (PBS), and lysed in radioimmuoprecipitation assay (RIPA) lysis buffer (Thermo Scientific, Waltham, MA, USA) containing Halt phosphotase and protease inhibitors (Thermo Scientific, Waltham, MA, USA). Equal amounts of protein were separated by sodium dodecyl sulfate (SDS)-polyacrylamide gel electrophoresis. Western blot analysis was performed as described previously [33,35].

### 2.5. Animal Models

All animal studies were carried out under protocols approved by the Institutional Animal Care and Use Committee at City of Hope (IACUC 11013). MDA-MB-468 cells (5 × 10^6^ in Matrigel) and 4T1 (1 × 10^5^) were inoculated into the mammary fat pad of 6- to 8-week-old female NOD/SCID/IL2Rgamma null (NSG) mice (MDA-MB-468) or Balb/C mice (4T1). Once the tumors were palpable, animals were randomized into four groups with 7–10 mice for each group to achieve an equal distribution of tumor sizes in all treatment groups. Mice were then treated with a vehicle, eribulin (retro-orbital), everolimus (oral gavage), or a combination of both agents. Tumor sizes were assessed using calipers once to twice a week. Tumor volumes were calculated using the formula Width^2^ × Length × 0.52. Body weight was monitored weekly as an indicator of the overall health of mice.

### 2.6. Immunohistochemistry (IHC)

Tumor tissues were fixed in 10% buffered formalin and embedded in paraffin. IHC was performed by the Pathology Core at City of Hope using VENTANA Ultra IHC automated stainer (VENTANA Medical Systems, Roche Diagnostics, Indianapolis, IN, USA). Briefly, tissue samples were sectioned at a thickness of 5 μm and put on positively charged glass slides. The slides were loaded on the machine and followed by deparaffinization, rehydration, endogenous peroxydase activity inhibition, and antigen retrieval. The slides were first incubated with primary antibody against Ki67 (Clone 30-9, Roche Diagnostics, Indianapolis, IN, USA) for proliferation and cleaved-caspase 3 (clone ASP175, Cell Signaling, Danvers, MA, USA) for apoptosis, and then incubated with DISCOVERY anti-Rabbit HQ and anti-HQ-HRP, visualized with the DISCOVERY ChromoMap DAB Kit, and counterstained with haematoxylin (Roche Diagnostics, Indianapolis, IN, USA). The immunoreactivity is evident as a dark brown color. Slides were scanned with VENTANA iScan HT using VENTANA Image Viewer (VENTANA Medical Systems, Roche Diagnostics, Indianapolis, IN, USA). The images were taken at 40x magnification.

### 2.7. Statistical Analysis

Data are presented as the mean ± S.D. A comparison of the means of two groups was determined by a Student’s t-test. Each experiment was carried out in triplicate or more. *p* values less than 0.05 were considered statistically significant.

## 3. Results

### 3.1. Eribulin Inhibits the Phosphorylation of AKT in Triple Negative Breast Cancer Cells

**Figure 1:**
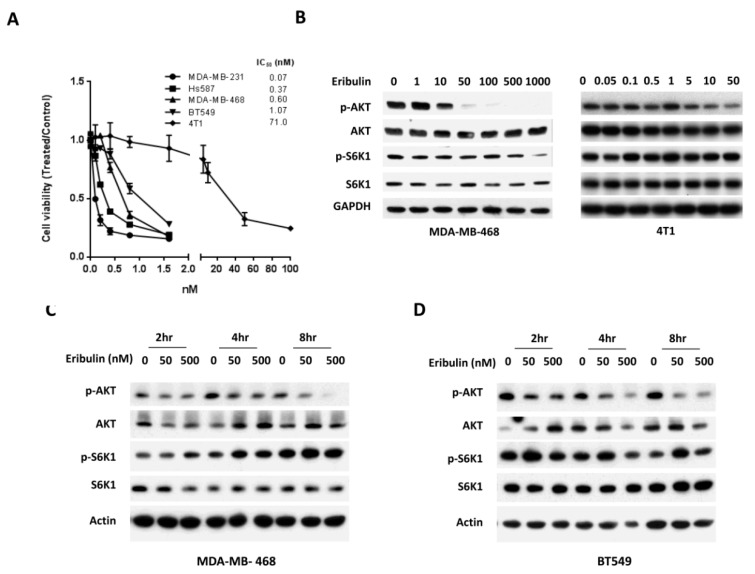
Eribulin inhibits cell viability and AKT phosphorylation in triple negative breast cancer (TNBC) cells. (**A**) TNBC cells were treated with various concentrations of eribulin. Cell viability was determined 72 h later. The IC_50_ was determined by the Chou-Talalay method. (**B**) Cells were treated with eribulin at concentrations of 1–1000 nM for MDA-MB-468 and 0.05–50 μM for 4T1 cells. Cells were harvested at 24 h and measured for the expression of p-AKT, AKT, p-S6K1, and S6K1 by Western blot analysis. (**C**–**D**) MDA-MB-468 and BT549 cells were treated with eribulin for the indicated times and concentrations. Cells were collected and measured for the expression of p-AKT and p-S6K1 by Western blot analysis.

We first studied the anti-tumor activity of eribulin in several TNBC lines. Cells were incubated with serial dilutions of eribulin. Cell viability was determined 72 h later. As shown in Figure 1A, eribulin inhibited cell viability, with an IC_50_ ranging from 0.07 to 71 nM in TNBC.

Activation of the PI3K/AKT pathway by some anti-cancer drugs has been previously shown to cause drug resistance [36]. To study the effect of eribulin on the PI3K/AKT pathway, MDA-MB-468 and 4T1 breast cancer cells were incubated with increasing concentrations of eribulin for 24 h, followed by Western blot analysis. We found that eribulin significantly decreased p-AKT expression in a dose-dependent manner (Figure 1B). The reduced expression of p-AKT by eribulin was seen as early as 4 h in both MDA-MB-468 and BT549 cells (Figure 1C,D).

**Figure 2:**
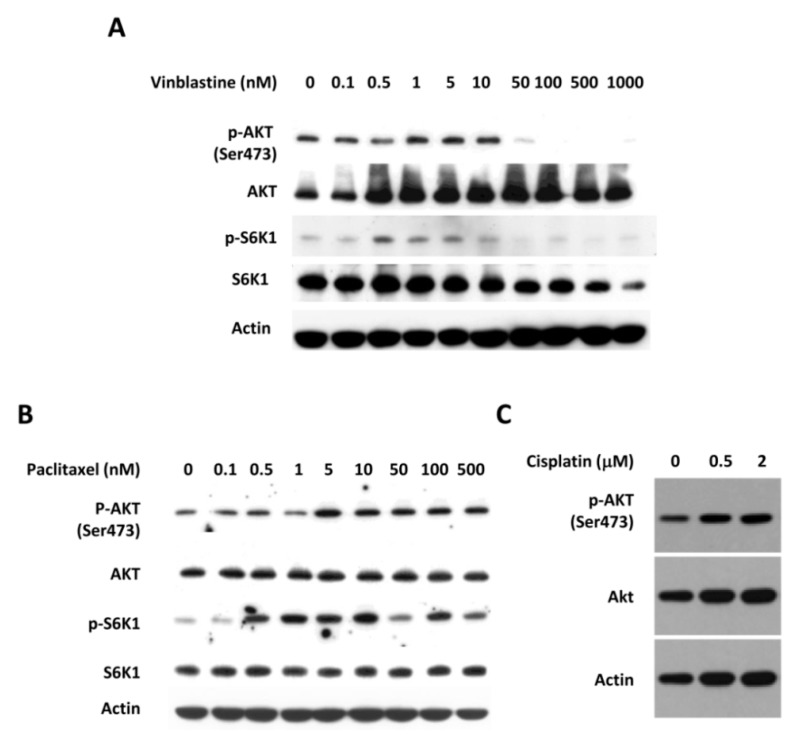
The effect of commonly used cytotoxic agents on AKT phosphorylation. MDA-MB-468 cells were treated with vinblastine (**A**), paclitaxel (**B**), and cisplatin (**C**) at indicated concentrations. Cells were harvested at 24 h, and the expression of p-AKT and p-S6K1 was measured by Western blot analysis.

We next compared the effect of eribulin on the PI3K/AKT pathway with two other microtubule targeting agents, vinblastine and paclitaxel, as well as a conventional DNA damage chemotherapeutic agent, cisplatin. Treatment with vinblastine, a microtubule depolymerizing agent similar to eribulin, resulted in a dose-dependent decrease in p-AKT expression in MDA-MB-468 cells. Treatment with paclitaxel, a microtubule stabilizing agent, resulted in a dose-dependent increase in p-AKT expression. Incubation of cisplatin with MDA-MB 468 also resulted in a dose-dependent increase in p-AKT expression in MDA-MB-468 cells (Figure 2).

Taken together, these results showed that p-AKT expression was suppressed in the presence of microtubule targeting agents that block tubulin polymerization, such as eribulin and vinblastine, in TNBC.

### 3.2. Combined Treatment of Eribulin and Everolimus Enhances the Reduction of p-S6K1 and p-S6

**Figure 3:**
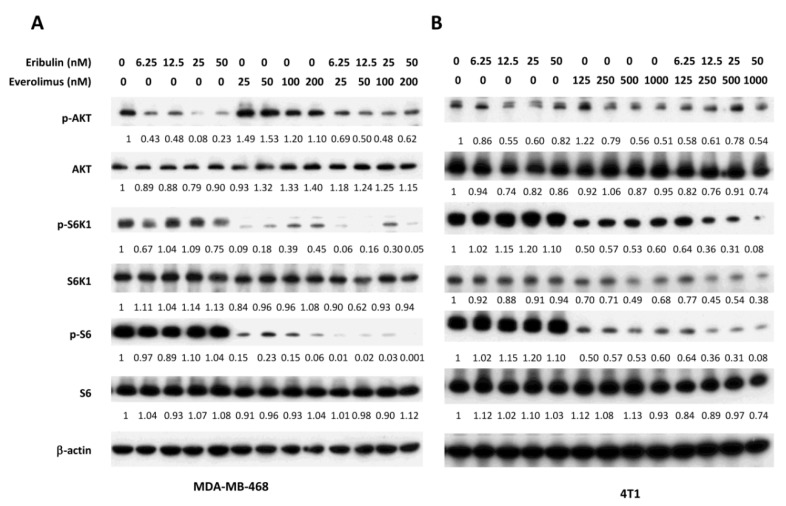
Combined treatment of eribulin and everolimus enhances the reduction of p-S6. MDA-MB-468 (**A**) and 4T1 (**B**) cells were treated with eribulin and everolimus at indicated concentrations, either alone or in combination. Cells were collected 24 h later and analyzed by Western blot for the expression of p-AKT, p-S6K, and p-S6. Numbers below the corresponding blot represent densitometric analysis normalized to β-actin.

Given the capability of eribulin to inhibit p-AKT and tumor growth, we next studied the benefit of combining eribulin with everolimus in TNBC. Everolimus, an inhibitor of mTOR, has emerged as a potential combination therapy drug for cancer treatment, although everolimus alone only exerts modest anti-cancer effects. Everolimus often increases the expression of p-AKT in human cancer cells when used alone. To investigate the effect of combined treatment of everolimus and eribulin on the PI3K/AKT/mTOR pathway, we incubated MDA-MB-468 cells with eribulin and everolimus at various concentrations, either alone or in combination. As shown in Figure 3, Western blot analysis for MDA-MB-468 cells treated with the combination of eribulin and everolimus showed a dose-related suppression of p-AKT expression, along with a greater inhibition of p-S6K1 and p-S6 expression. Combination treatment also caused a greater inhibition of p-S6K1 and p-S6 in 4T1, a highly metastatic mouse TNBC cell line.

### 3.3. Combined Treatment of Eribulin and Everolimus Synergistically Inhibits Cell Viability

**Figure 4:**
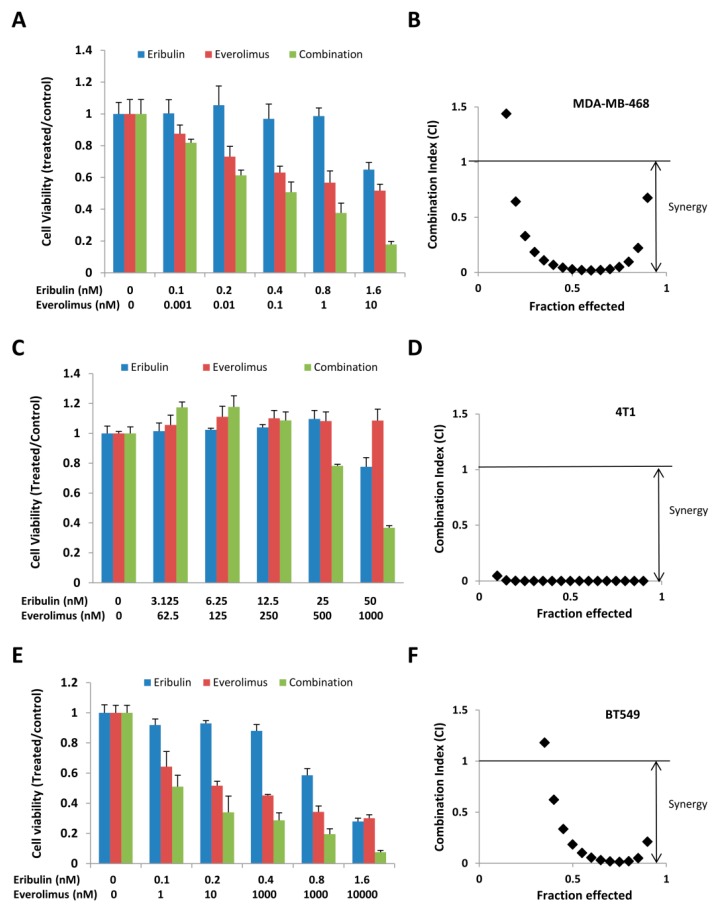
Combined treatment of eribulin and everolimus resulted in synergistic cell growth inhibition. MDA-MB-468 (**A**,**B**), 4T1 (**C**,**D**), and BT549 (**E**,**F**) cells were treated with eribulin or everolimus, either alone or in combination, at indicated concentrations. Cell viability was determined 72 h later. The combination index (CI) was calculated by the Chou-Talalay method.

We next evaluated whether the combination of eribulin and everolimus resulted in more effective ant-tumor activity. To address this, MDA-MB-468, 4T1, and BT549 cells were treated with eribulin and everolimus, either alone or in combination. Cell viability was determined 72 h later. The combination treatment decreased cell viability more robustly than either agent alone (Figure 4). To determine whether the increased activity was additive or synergistic, the combination index (CI) was calculated according to the Chou-Talalay method. As shown in Figure 4, the combined treatment of erubulin and everolimus caused very strong synergism in all three cell lines.

### 3.4. Combination of Eribulin with PI3K Inhibitors, BEZ 235 and BKM 120, has a Similar Effect on p-S6K/p-S6 and Cell Viability

**Figure 5:**
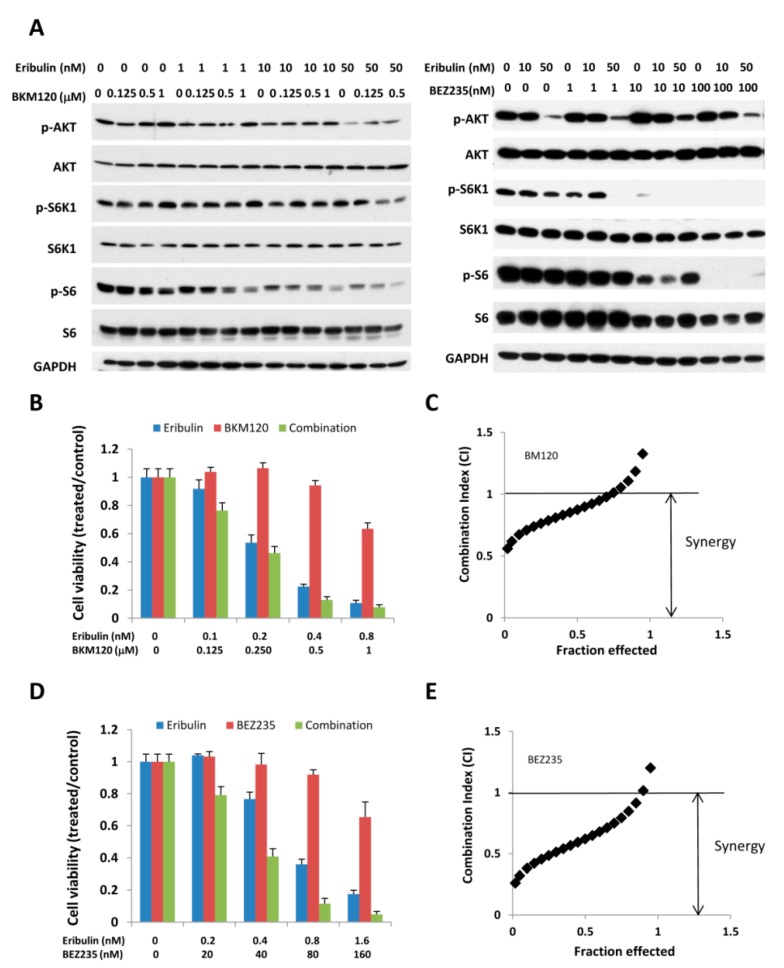
Combined treatment of eribulin with BKM120 and BEZ 235 enhances the inhibition of cell growth. (**A**) MDA-MB-468 cells were treated with eribulin, BKM120, or BEZ 235, either alone or in combination, at indicated concentrations. Cells were collected 24 h later and measured for p-AKT, p-S6K, and p-S6 expression. (**B**–**E**) MDA-MB-468 cells were treated with eribulin, BKM120 (**B**,**C**), or BEZ235 (**D**,**E**), either alone or in combination, at indicated concentrations. Cell viability was determined 72 h later (**B**,**D**). The combination index (CI) was determined according to the Chou-Talalay method (**C**,**E**).

Because the combination of eribulin with everolimus enhances anti-tumor activity, we next asked whether a combination of eribulin and other PI3K/AKT/mTOR inhibitors could achieve a similar result. To address this, we combined eribulin with BEZ235 or BKM120, two pan-class PI3K/AKT/mTOR inhibitors that induce pathway inhibition and show anti-tumor activity in PI3K-pathway-dysregulated cancers. As shown in Figure 5A, the combination of eribulin with BEZ235 or BKM120 was more effective in inhibiting the expression of p-AKT, p-S6K1, or p-S6 than either agent alone. We also evaluated whether combination treatment increases anti-tumor activity. MDA-MB-468 cells were incubated with eribulin at various concentrations, either alone or in combination with BEZ235 or BKM120. Cell viability was determined 72 h later. As shown in Figure 5B–5E, the combination of eribulin with either BEZ235 or BKM120 synergistically inhibits growth. Taken together, our study shows significant synergistic growth inhibition when eribulin is combined with PI3K/AKT/mTOR inhibitors.

### 3.5. Combined Treatment of Eribulin and Everolimus Enhances Anti-tumor Activity in Mice

**Figure 6:**
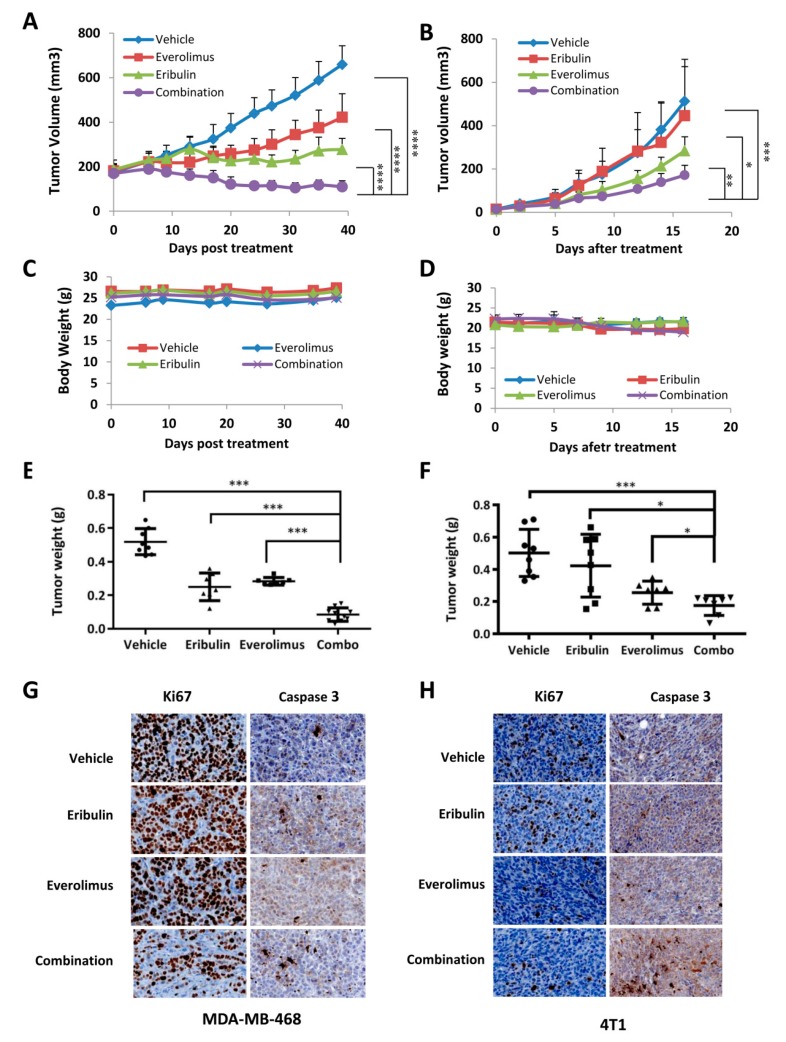
Combined treatment of eribulin and everolimus enhances anti-tumor activity in mice. MDA-MB-468 (**A**,**C**,**E**,**G**) and 4T1 (**B**,**D**,**F**,**H**) were implanted into the mammary fat pad of NOD/SCID/IL2Rgamma null (NSG) mice and BALB/c mice, respectively. NSG mice with MDA-MB-468 tumors were treated with a vehicle, eribulin (0.2 mg/kg for the first week, 0.1 mg/kg for the remainder of the treatment period, via retro-orbital), everolimus (5 mg/kg via oral gavage), or their combination two times a week. BALB/c mice with 4T1 tumors were treated with a vehicle, eribulin (1 mg/kg via retro-orbital), everolimus (5 mg/kg via oral gavage), or their combination three times a week. Tumor growth (**A**,**B**) and body weight (**C**,**D**) were measured once to twice a week. (**E**,**F**) Tumor weight was measured at end of the treatment (**G**,**H**) Shown are representative images of Ki67 and cleaved-caspase 3 immunohistochemstry in MDA-MB-468 and 4T1 tumor tissues. Data represents means ± SD (n = 7–10). *, *p* < 0.05; **, *p* < 0.005. ***, *p* < 0.0005, ****, *p* < 0.0001.

Next, we investigated whether the combination treatment could suppress tumor growth in vivo more effectively than either treatment alone. Mammary fat pads of NSG mice and BALB/c mice were inoculated with MDA-MB-468 human breast cancer cells and 4T1 mouse breast cancer cells, respectively. MDA-MB-468 is a human TNBC cell line. 4T1 is a highly metastatic TNBC cell line derived from a spontaneously arising BALB/c mammary tumor. When the tumors were palpable, we randomized mice into four treatment groups (eribulin, everolimus, eribulin plus everolimus, or vehicle control). No severe toxicity was observed in mice treated with the combination (Figure 6C,D). Treatment with eribulin and everolimus alone had a modest anti-tumor effect. However, the combination of eribulin and everolimus was more effective than any single treatment in both the MDA-MB-468 tumor and 4T1 tumor (Figure 6).

Taken together, our study demonstrates that the combination of eribulin plus everolimus markedly enhances the suppression of tumors compared to treatment with eribulin or everolimus alone in two mouse models of TNBC: a syngeneic model with a well-known highly metastatic TNBC (4T1) and a xenogeneic model with human TNBC (MDA-MB-468). These findings indicate a potential role for eribulin/everolimus combination therapy in the treatment of refractory TNBC.

## 4. Discussion

Despite advances in breast cancer treatment, patients with TNBC have worse outcomes after chemotherapy than patients with other subtypes of breast cancer [1]. In the era of personalized cancer therapy, molecular characteristics have been sought to identify new therapeutic targets [5,6]. Efforts to profile TNBC tumors have revealed the PI3K/AKT/mTOR pathway as a potential therapeutic target. Activation of this pathway also contributes to the resistance to anti-cancer agents, including microtubule-targeting agents. In this study, we show, for the first time, that the phosphorylation of AKT is suppressed by microtubule depolymerizing agents, eribulin and vinblastine, but activated by microtubule stabilizing agents, such as paclitaxel, or by a conventional DNA damaging chemotherapeutic agent, cisplatin. Dual treatment of eribulin and everolimus results in an increased reduction of p-S6K1 and p-S6, a synergistic suppression of cell survival in a number of breast cancer cell lines in vitro, and an enhanced suppression of tumor growth in two breast cancer mouse models.

Eribulin mesylate is a microtubule-targeting agent used to treat taxane and anthracycline refractory breast cancer [18,19,20,21,22,23,24,29]. Phase I clinical trials of eribulin in patients with previously treated solid malignancies demonstrated a dose escalation response that was limited by neutropenia and fatigue. In these trials, eribulin had linear pharmacokinetics with a rapid distribution phase, extensive volume distribution with slow to moderate clearance, and slow elimination [37,38,39]. Phase II clinical trials were conducted in patients with heavily pretreated metastatic breast cancer. In these trials, eribulin exhibited antitumor activity with a manageable tolerability profile, with side effects consisting of neutropenia, fatigue, alopecia, nausea, and anemia. In addition, there was a low incidence of peripheral neuropathy [40,41,42]. In the Eisai Metastatic Breast Cancer Study Assessing Physician’s Choice Verses E7389 clinical trial, a phase III trial of patients with heavily pretreated metastatic breast cancer, participants received eribulin monotherapy or treatment of physician’s choice (TPC). Enrolled patients had received a median of four prior therapies. Improvement was seen in overall survival (OS) with hazard ratio (HR) 0.81 (95% CI: 0.66–0.99, *p* = 0.041). Median OS was 13.1 months in patients receiving eribulin versus 10.6 months in TPC [29]. This study led to the FDA approval of eribulin mesylate for the treatment of breast cancer in patients who had failed taxane- or anthracycline-based therapies.

The PI3K/AKT/mTOR signaling pathway has been implicated in the regulation of microtubule stability by growth factors and drug resistance [43]. In this study, we compared the ability of eribulin to inhibit the PI3K/AKT/mTOR pathway and cell growth with that of two other microtubule-targeting agents, paclitaxel and vinblastine. We found that both eribulin and vinblastine decreased the expression of p-AKT in TNBC cells. Growth inhibition was also seen with treatment with eribulin or vinblastine. Interestingly, treatment of TNBC with paclitaxel or the conventional chemotherapeutic cisplatin resulted in an increased expression of PI3K downstream proteins. Although the mechanism by which eribulin inhibits the phosphorylation of AKT remains to be elucidated, it is possible that depolymerization of the microtubule may interfere with the localization of AKT in the cells. It has been previously shown that the localization of AKT to microtubules is important for sustaining AKT phosphorylation [44,45].

Eribulin was found to inhibit cell growth in MDA-MB-435 triple negative cancer cells at a lower concentration than paclitaxel or vinblastine in vitro and suppressed 95% of growth in breast cancer xenografts in vivo [20]. In a study of paclitaxel-resistant human cancer cells in vitro, eribulin and vinblastine maintained their full potency to inhibit cell proliferation [46]. Enhancement of AKT activity is likely a survival response by cancer cells to chemotherapy, yet AKT activity appears to be suppressed when microtubule polymerization is blocked. These findings suggest a mechanism through which eribulin inhibits the cell growth of refractory triple negative and HER2 expressing breast cancer.

Resistance to microtubule-targeting agents may be due to a multidrug resistant phenotype or the activation of growth signaling pathways [32]. A better understanding of the mechanisms behind drug resistance and the call for more personalized medicine have sparked interest in combination therapy regimens aimed at multiple targets. Drug combination aims to decrease the drug dose and toxicities, achieve a synergistic effect, or overcome drug resistance [34]. Loss of the tumor suppressor PTEN (phosphatase and tensin homolog) and activation of the PI3K pathway have been implicated in resistance to endocrine therapy and trastuzumab [11,47,48,49,50]. PTEN works to antagonize the PI3K pathway activation of downstream targets AKT and mTOR. When PTEN is lost, the pathway goes unregulated, resulting in enhanced tumorigenesis [49,51]. The downstream PI3K inhibitors can be used to overcome the drug resistance.

Everolimus, an inhibitor of mTOR, has emerged as a potential combination therapy drug for the treatment of cancer that does not respond to conventional therapy [52]. When used alone, everolimus can induce increased levels of p-AKT via a negative feedback loop, leading to the resistance of cells to mTOR inhibitors [13,14]. A dual blockade of mTOR and other PI3K pathway inhibitors results in synergistic decreases in cancer cell growth [13,14,53,54]. Our findings that eribulin treatment decreases activation of the PI3K/AKT/mTOR pathway led us to investigate the possible synergy between eribulin and everolimus. Our results demonstrate that dual treatment of eribulin and everolimus increases the reduction of p-S6K1 and p-S6 expression, a synergistic suppression of cell survival in vitro, and an enhanced suppression of tumor growth in mouse models. Therefore, targeting both the microtubule cytoskeleton and the PI3K/AKT/mTOR pathway can lead to a synergistic anti-tumor effect.

The combination of everolimus with endocrine therapy was effective in the treatment of hormone receptor positive breast cancers. A phase III clinical trial in patients with hormone receptor positive metastatic breast cancer previously treated with aromatase inhibitors demonstrated that combination therapy with exemestane plus everolimus showed improvement in progression-free survival (HR for progression or death=0.43; 95% CI: 0.35 to 0.54; *p* < 0.001) versus exemestane alone [55,56]. This led to the FDA approval of everolimus for advanced or metastatic aromatase inhibitor-resistant ER + breast cancer [57]. The combination of tamoxifen and everolimus was active in hormone receptor positive breast cancer [58]. In addition, the efficacy of everolimus in Her2-neu overexpressed breast cancer was also confirmed in a phase I/II clinical trial of trastuzumab plus everolimus, with a clinical benefit rate of 34% [59]. These findings from the clinical trials suggest a possible mechanism of drug resistance through continued PI3K/AKT/mTOR pathway activation and that combination treatment with everolimus may re-sensitize cancer to the targeted drug. The efficacy of the combination of everolimus and carboplatin was tested in a phase I trial with a tolerable safety profile and modest clinical activity [60]. An ongoing clinical trial is comparing the combination of carboplatin and everolimus versus carboplatin alone (NCT02531932).

## 5. Conclusions

We have demonstrated preclinical results to support the use of the microtubule-targeting agent, eribulin, in combination with the mTOR inhibitor, everolimus, against TNBC. This combination therapy is currently being tested in a phase I clinical trial in patients with TNBC who have progressed on anthracyclines and/or taxanes (NCT02120469).
